# SMAC mimetics induce autophagy-dependent apoptosis of HIV-1-infected macrophages

**DOI:** 10.1038/s41419-020-02761-x

**Published:** 2020-07-27

**Authors:** Grant R. Campbell, Rachel K. To, Gang Zhang, Stephen A. Spector

**Affiliations:** 1 Division of Infectious Diseases, Department of Pediatrics, University of California San Diego, La Jolla, CA USA; 2 Rady Children’s Hospital, San Diego, CA USA; 3 Present Address: Chicago Medical School, Rosalind Franklin University of Medicine and Science, North Chicago, IL USA; 4 Present Address: Gladstone Center for HIV Cure Research, Gladstone Institute of Virology and Immunology, San Francisco, CA USA

**Keywords:** Apoptosis, Necroptosis, HIV infections

## Abstract

Human immunodeficiency type 1 (HIV)-infected macrophages (HIV-Mφ) are a reservoir for latent HIV infection and a barrier to HIV eradication. In contrast to CD4+ T cells, HIV-Mφ are resistant to the cytopathic effects of acute HIV infection and have increased expression of cell survival factors, including X-linked inhibitor of apoptosis (XIAP), baculoviral IAP repeat containing (BIRC) 2/cIAP1, beclin-1, BCL2, BCL-xl, triggering receptor expressed on myeloid cells 1, mitofusin (MFN) 1, and MFN2. DIABLO/SMAC mimetics are therapeutic agents that affect cancer cell survival and induce cell death. We found that DIABLO/SMAC mimetics (LCL-161, AT-406 (also known as SM-406 or Debio 1143), and birinapant) selectively kill HIV-Mφ without increasing bystander cell death. DIABLO/SMAC mimetic treatment of HIV-Mφ-induced XIAP and BIRC2 degradation, leading to the induction of autophagy and the formation of a death-inducing signaling complex on phagophore membranes that includes both pro-apoptotic or necroptotic (FADD, receptor-interacting protein kinase (RIPK) 1, RIPK3, caspase 8, and MLKL) and autophagy (ATG5, ATG7, and SQSTM1) proteins. Genetic or pharmacologic inhibition of early stages of autophagy, but not late stages of autophagy, ablated this interaction and inhibited apoptosis. Furthermore, DIABLO/SMAC mimetic-mediated apoptosis of HIV-Mφ is dependent upon tumor necrosis factor signaling. Our findings thus demonstrate that DIABLO/SMAC mimetics selectively induce autophagy-dependent apoptosis in HIV-Mφ.

## Introduction

Although latently infected CD4+ T cells are the predominant human immunodeficiency virus type 1 (HIV) reservoir in infected persons on suppressive antiretroviral therapy (ART), other cell types including macrophages serve as important sites of viral persistence^1–3^. Long-lived HIV-infected perivascular macrophages contain replication-competent proviral HIV DNA, support persistent permissive HIV infection in the absence of CD4+ T cells^4,5^, and contain lower intracellular concentrations of antiretrovirals than CD4+ T cells resulting in ongoing HIV replication^6^. HIV-infected macrophages also harbor and synaptically transmit replication-competent virus to nearby CD4+ T cells, a process that is resistant to antibody-mediated neutralization^7^. Additionally, HIV signals through multiple pathways to reprogram the transcriptome and proteome of human macrophages rendering them resistant to both the cytopathic effects of HIV infection and to CD8+ T-cell-mediated killing^8–10^. HIV decreases the expression of pro-apoptotic proteins, while increasing the expression of anti-apoptosis regulatory proteins, including BCL2 family members, and the inhibitor of apoptosis proteins (IAP) X-linked inhibitor of apoptosis (XIAP), baculoviral IAP repeat containing (BIRC) 2 (formerly known as cIAP1), and BIRC3 (refs. ^11–21^). The overexpression of IAPs can inhibit cell death through a number of mechanisms, including direct inhibition of caspases and ubiquitination of receptor-interacting protein kinase (RIPK) 1, an important convergence point between pro-death, pro-survival, and proinflammatory signals. They may also play a role in the establishment of HIV latency and cytopathogenesis^22^. Thus, IAP are relevant targets for therapeutic exploitation in HIV cure strategies.

To circumvent the ability of IAP to turn pro-death into pro-survival signals in cancer cells, small-molecule DIABLO/SMAC peptidomimetics (SM) were developed that mimic the N-terminal 4 amino acid IAP-binding sequence of DIABLO. The interaction of SM with XIAP leads to caspase activation^23^, while the binding of SM to BIRC2 and BIRC3 enhances their E3 ligase activity promoting their autoubiquitination and proteasomal degradation with subsequent cell death^19,24,25^. In this study, we examined the ability of the SM LCL-161 (ref. ^26^), AT-406 (ref. ^27^), and birinapant^28^ to induce selective cell death in HIV-infected macrophages.

## Results

### DIABLO mimetics selectively kill HIV-infected macrophages

**Fig. 1:**
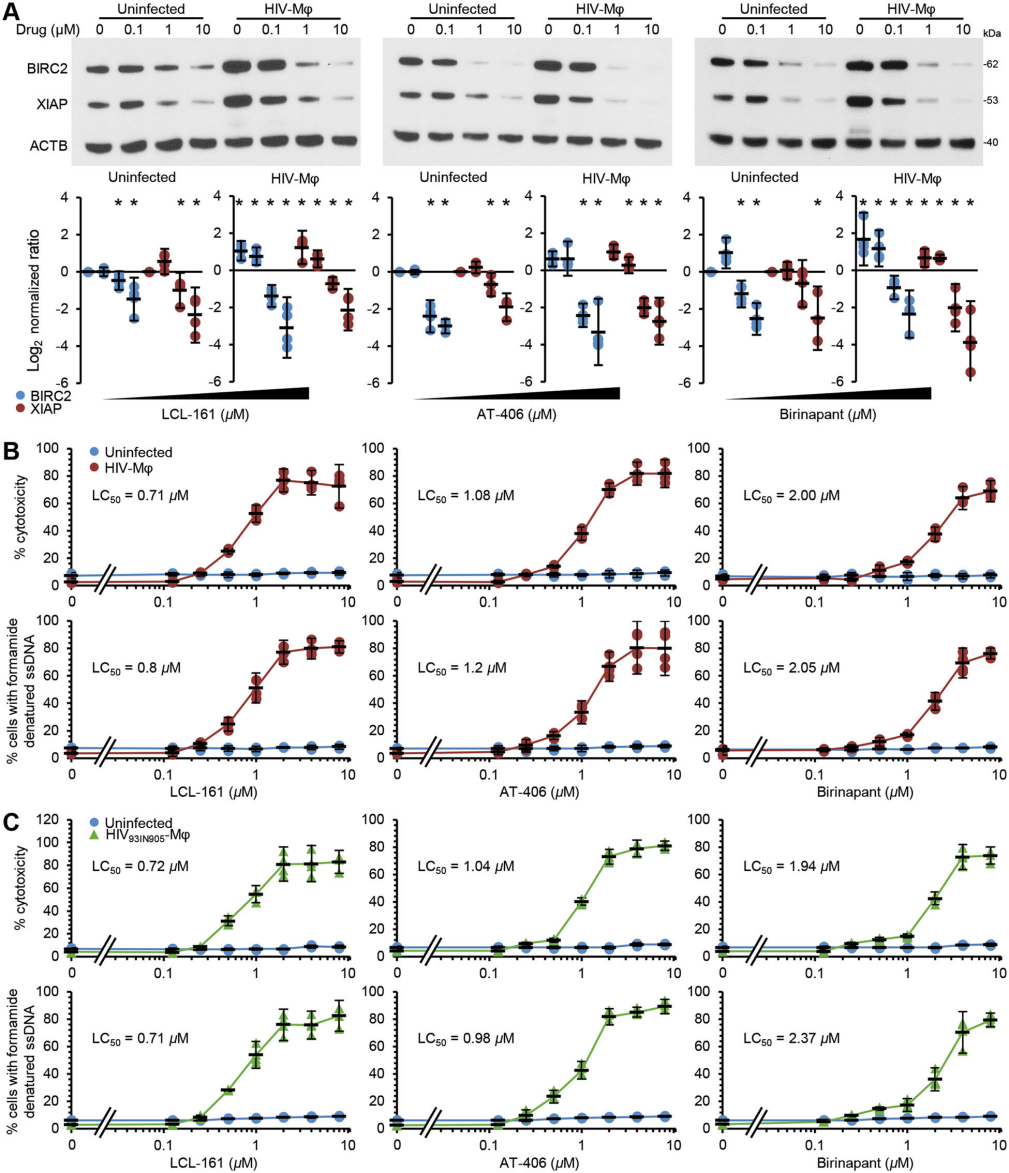
DIABLO/SMAC mimetics induce the degradation of BIRC2 and XIAP, and preferentially induce cell death in HIV-infected macrophages. **a** Uninfected macrophages and HIV_Ba-L_-infected macrophages (HIV-Mφ) were treated for 48 h with SM. Top, representative western blots of BIRC2, XIAP, and ACTB. Bottom, densitometric analysis of blots, *n* = 4. **b** Uninfected macrophages and HIV-Mφ were treated for 48 h with SM. Top, aliquots of supernatants were spectrophotometrically tested for LDH using the LDH^PLUS^ assay. Bottom, cells were fixed and permeabilized, and the percentage of cells with apoptotic ssDNA damage was quantified by ELISA, *n* = 4. **c** Uninfected macrophages and HIV_93IN905_-infected macrophages were treated for 48 h with SM. Top, aliquots of supernatants were spectrophotometrically tested for LDH using the LDH^PLUS^ assay. Bottom, cells were fixed and permeabilized, and the percentage of cells with apoptotic ssDNA damage was quantified by ELISA, *n* = 4.

We previously showed that HIV latently infected CD4+ T cells (HIV-T_CM_) have increased expression of BIRC2 and XIAP^19^. Therefore, we examined the expression of BIRC2 and XIAP in HIV_Ba-L_-infected macrophages (HIV-Mφ; Supplementary Fig. 1); XIAP and BIRC2 expression was significantly upregulated in HIV-Mφ (*P* < 0.05; Fig. 1). As SM induce the rapid degradation of BIRC2 and XIAP in cancer cells and HIV-T_CM_^19,24,25,29–31^, we evaluated the ability of the SM LCL-161, AT-406, and birinapant to induce BIRC2 and XIAP degradation in uninfected macrophages and HIV-Mφ. All three compounds induce significant BIRC2 and XIAP degradation in uninfected cells and HIV-Mφ (Fig. 1a). However, the concentrations required to induce significant degradation are 10–100× lower in HIV-Mφ than in uninfected cells.

**Fig. 2:**
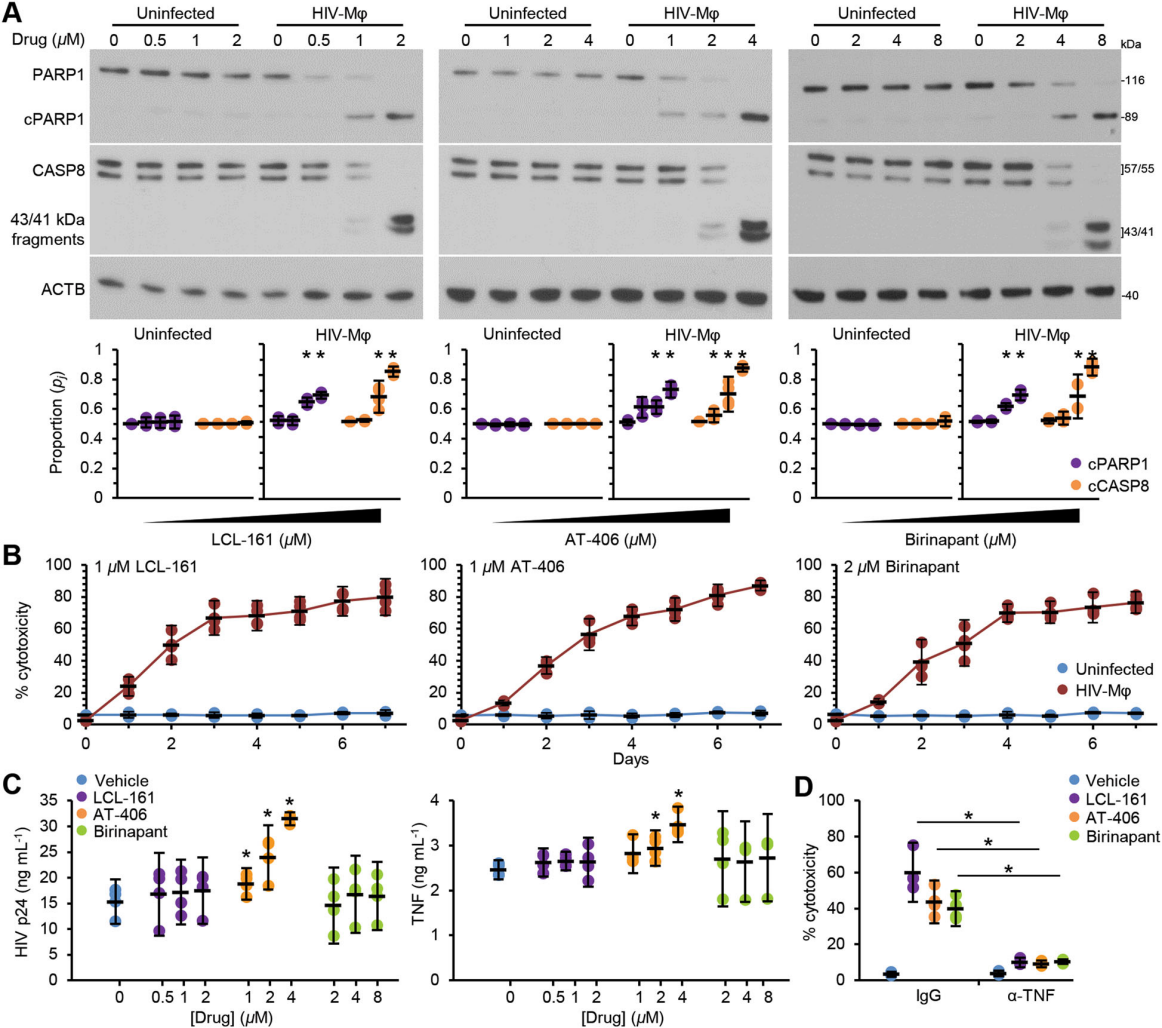
DIABLO/SMAC mimetics preferentially induce cell death in HIV-infected macrophages. **a** Uninfected macrophages and HIV-Mφ were treated with SM for 48 h. Top, representative western blots of PARP1 and CASP8 cleavage (cPARP1 and cCASP8), using antibody to PARP1, CASP8, and ACTB. Bottom, densitometric analysis of blots, *n* = 4. **b** Uninfected macrophages and HIV-Mφ were treated for 168 h with SM. Over time, aliquots of supernatants were spectrophotometrically tested for LDH using the LDH^PLUS^ assay, *n* = 4. **c** ELISA performed for HIV p24 antigen and TNF in supernatants from cells treated in **a**, *n* = 4. **d** HIV-Mφ were pretreated with IgG control or TNF neutralizing antibody 2 h before incubation with 1 µM LCL-161, 2 µM AT-406, or 4 µM birinapant for 48 h. Aliquots of supernatants were spectrophotometrically tested for LDH using the LDH^PLUS^ assay, *n* = 4.

SM-induced rapid degradation of BIRC2 and XIAP is a key early event in SM-induced cell death^24,25,29–31^. We evaluated the toxicity of the three SM against both uninfected macrophages and HIV-Mφ, using the presence of formamide-sensitive single-stranded DNA (a specific indicator of apoptosis^32^) and the release of lactate dehydrogenase (LDH). In uninfected macrophages, LCL-161, AT-406, and birinapant all showed minimal toxicity at the concentrations tested (Fig. 1b). Conversely, all three SM induced a dose-dependent increase in HIV-Mφ cytotoxicity. We observed similar results in HIV_93IN905_-infected macrophages (Fig. 1c). The increase in HIV-Mφ cytotoxicity corresponded to the dose-dependent proteolysis of poly(ADP-ribose) polymerase 1 (PARP1; a substrate of caspase (CASP) 3) and CASP8 cleavage (Fig. 2a). We then assessed whether the kinetics of killing uninfected macrophages is slower than that observed in HIV-Mφ. We observed no significant increase in LDH release in the uninfected cells post-SM treatment over 168 h, indicating that at no point do they undergo biologically significant cell death in response to SM (Fig. 2b). In contrast, SM induced a time-dependent increase in HIV-Mφ cytotoxicity. Importantly, we measured no SM-mediated increase in HIV p24 antigen release after LCL-161 or birinapant treatment, indicating that the SM were killing HIV-Mφ in the absence of increased virus production (Fig. 2c). Conversely, AT-406 treatment induced a dose-dependent increase of HIV p24 antigen release.

SM-mediated cell death conventionally requires the presence of pro-apoptotic tumor necrosis factor (TNF) family ligands^33,34^, and we previously demonstrated that SM selectively induce TNF-independent death of HIV-T_CM_^19^. Therefore, to determine the role of TNF in SM-mediated death of HIV-Mφ, we blocked TNF signaling with a specific neutralizing antibody and observed that neutralization of TNF significantly ablated SM-mediated HIV-Mφ cell death (Fig. 2d), suggesting that, unlike HIV-T_CM_, the SM-mediated killing of HIV-Mφ is TNF dependent.

**Fig. 3:**
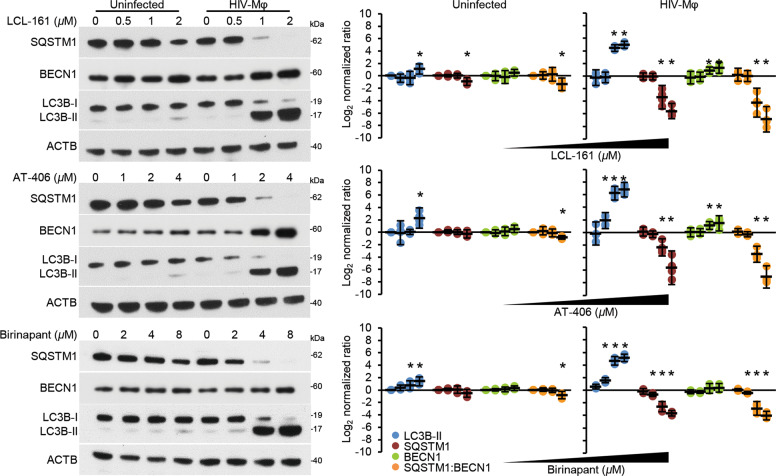
DIABLO/SMAC mimetics induce autophagy in HIV-infected macrophages. Uninfected macrophages and HIV-Mφ were treated for 48 h with SM. Left, representative western blots of LC3B isoforms, BECN1, and SQSTM1. Right, densitometric analysis of blots, *n* = 4.

**Fig. 4:**
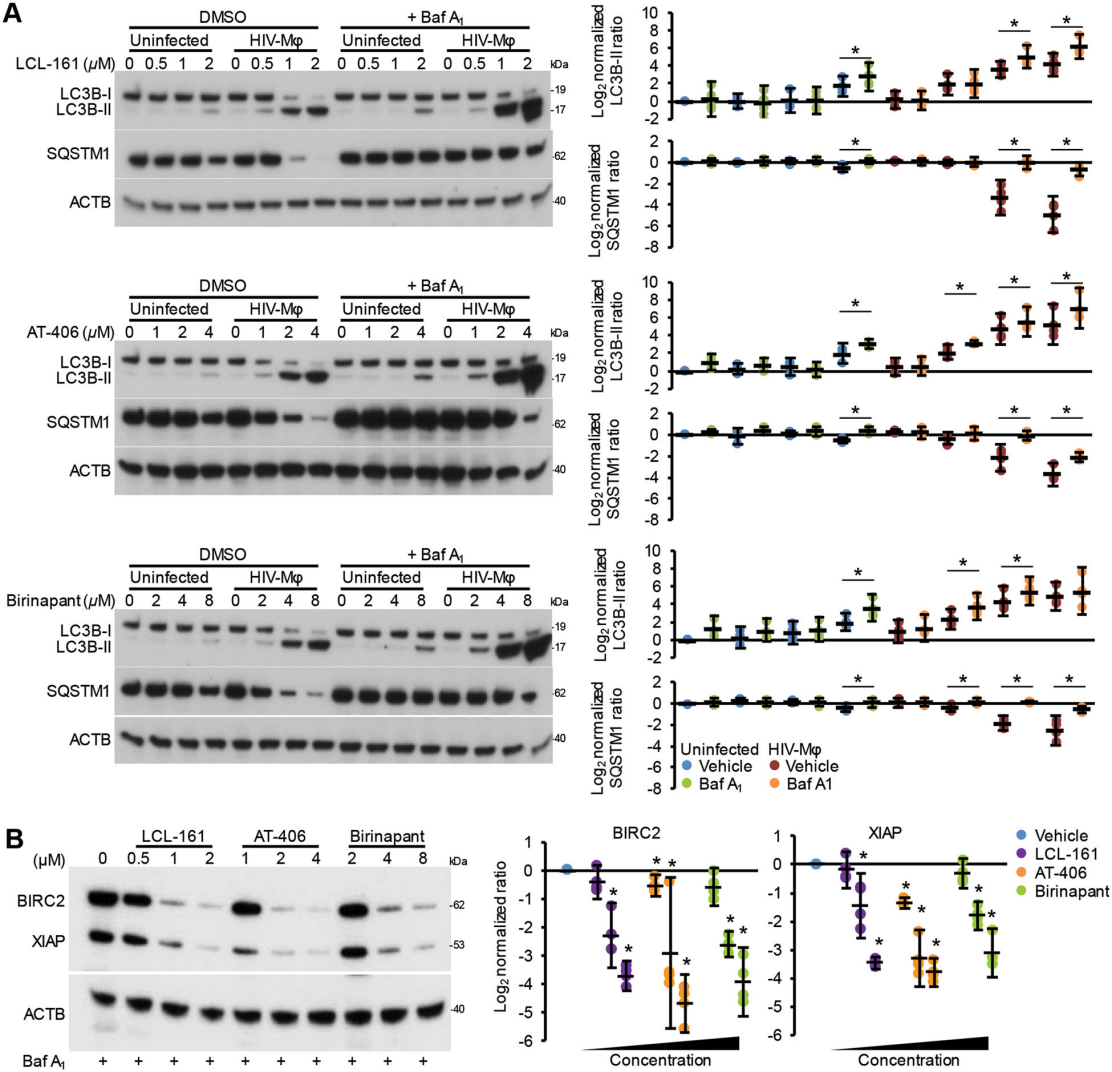
Autophagy induced by DIABLO/SMAC mimetics in HIV-infected macrophages goes to completion. Uninfected macrophages and HIV-Mφ were pretreated with bafilomycin A1 before treatment with SM for 48 h. **a** Left, representative western blots of LC3B isoforms, BECN1, and SQSTM1. Right, densitometric analysis of blots, *n* = 4. **b** Left, representative western blots of BIRC2 and XIAP. Right, densitometric analysis of blots, *n* = 4.

Although SM induce the proteasomal degradation of IAP, we examined whether they also stimulate autophagy in HIV-Mφ. Autophagic flux is assessed by monitoring the biogenesis of autophagosomes through a ubiquitin-like system that involves autophagy-related (ATG) 7 and the ATG12–ATG5 complex that converts cytosolic microtubule-associated protein 1 light chain 3 beta (MAP1LC3B or LC3B)-I to LC3B-II. The ATG12–ATG5 complex then ligates LC3B-II to the nascent autophagosome membrane. The polyubiquitin-binding protein sequestosome 1 (SQSTM1, p62) and SQSTM1-bound polyubiquitinated proteins are incorporated into completed autophagosomes that then fuse with lysosomes, resulting in the degradation of the engulfed components, as well as LC3B-II and SQSTM1 associated with the inner membrane. Thus, the quantification of SQSTM1 and the conversion of LC3B-I to LC3B-II, and its turnover are indicators of autophagy induction and flux^35^. In uninfected macrophages, SM increases LC3B lipidation at the highest concentrations tested while having no significant effect on SQSTM1 degradation, indicating an absence of induced autophagy (Fig. 3a). In contrast, LCL-161 and AT-406 induced a dose-dependent significant increase in LC3B-II lipidation, BECN1 expression, and SQSTM1 degradation. While we observed a change in LC3B lipidation and SQSTM1 expression using birinapant, at no point did we observe an increase in BECN1. To confirm the induction of autophagy in SM-treated HIV-Mφ, and not simply the activation of *MAP1LC3B* and *SQSTM1* transcription, we used bafilomycin A_1_, an inhibitor of autophagosome–lysosome fusion. Blots of cell lysates confirmed autophagic flux in HIV-Mφ, with increased LC3B-II and SQSTM1 accumulation in bafilomycin A_1_-treated cells relative to vehicle controls (Fig. 4). Crucially, pretreatment with bafilomycin A_1_ did not reduce the effect of SM-induced XIAP or BIRC2 degradation in HIV-Mφ (Fig. 4b).

### SM induce apoptosis in HIV-infected macrophages

**Fig. 5:**
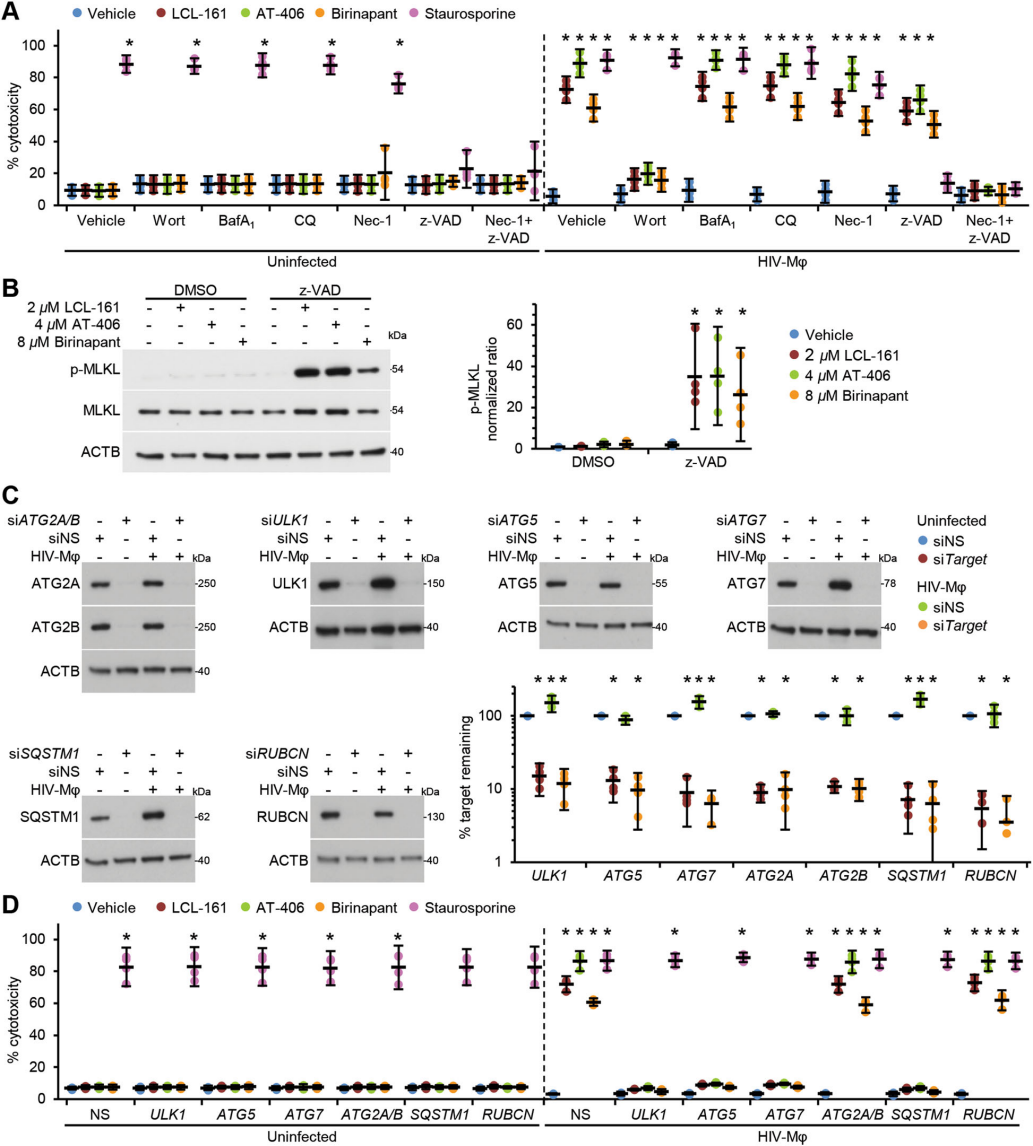
DIABLO/SMAC mimetics promote apoptosis in HIV-infected macrophages using an autophagy dependent mechansim. **a** Uninfected macrophages and HIV-Mφ with pretreated with wortmannin (Wort), bafilomycin A_1_ (BafA_1_), chloroquine (CQ), necrostatin-1 (Nec-1), z-VAD-FMK (z-VAD), or vehicle control (vehicle) for 2 h. Cell death was measured 48 h after subsequent 2 µM LCL-161, 4 µM AT-406, 8 µM birinapant, or 1 µM staurosporine using the LDH^PLUS^ assay, *n* = 4. **b** HIV-Mφ were pretreated with z-VAD or vehicle control (DMSO) for 2 h before treatment with SM. Left, representative western blots of phosphorylated MLKL (p-MLKL) and total MLKL (MLKL). Right, densitometric analysis of blots, *n* = 4. **c** Uninfected macrophages and HIV-Mφ simultaneously transfected with *ATG2A* and *ATG2B* siRNA (si*ATG2A/B*), *ATG5* siRNA (si*ATG5)*, *ATG7* (si*ATG7*), *RUBCN* (si*RUBCN*), *SQSTM1* (si*SQSTM1*), *ULK1* (si*ULK1*), or scrambled siRNA (siNS). Left, representative western blots of ATG2A, ATG2B, ATG5, ATG7, SQSTM1, ULK1, and RUBCN. Right, densitometric analysis of blots, *n* = 4. **d** Cells from **c** were incubated with 2 µM LCL-161, 4 µM AT-406, 8 µM birinapant, or 1 µM staurosporine for 48 h. Cell death was estimated using the LDH^PLUS^ assay, *n* = 4.

To determine the mechanism of cell death, we examined the effect of SM on HIV-Mφ in the presence of pharmacological inhibitors of apoptosis (pan-caspase inhibitor z-VAD-FMK), autophagy (PI3K inhibitor wortmannin, bafilomycin A_1_, and chloroquine, a lysosomotropic agent that prevents endosomal acidification), and/or necroptosis (RIPK1 inhibitor necrostatin-1). None of the HIV-Mφ tested showed a purely apoptotic or necroptotic phenotype in response to SM with neither z-VAD-FMK nor necrostatin-1 alone able to arrest SM-induced cell death (Fig. 5a). However, co-treatment with both z-VAD-FMK and necrostatin-1 resulted in an 88.9% reduction in SM-mediated death of HIV-Mφ. During necroptosis, the effector mixed-lineage kinase domain-like protein (MLKL) is recruited to the necrosome where it is phosphorylated by RIPK3, inducing a conformational change that causes it to translocate to and permeabilize the plasma membrane^36^. Therefore, we assessed the role for MLKL in SM-mediated cell death. In the absence of z-VAD-FMK, we did not observe significant SM-mediated MLKL phosphorylation despite observing 50–66% cell death (Fig. 5b). However, when we pretreated cells with z-VAD-FMK, SM treatment induced significant MLKL phosphorylation, suggesting that cells switched to necroptosis when we inhibited apoptosis.

We then examined how autophagy affects SM-mediated cell death in HIV-Mφ. In these experiments, inhibition of the autophagy conjugation cascades with wortmannin or RNA interference (RNAi) for *ULK1*, *ATG5*, *ATG7*, or *SQSTM1* (Fig. 5c) led to increased cell viability in the presence of SM (Fig. 5a, d). In contrast, we observed no effect on SM-induced death by blocking autophagic flux using either simultaneous RNAi for *ATG2A* and *ATG2B* (*ATG2A/B*), which causes the accumulation of immature unclosed autophagosomal structures (Fig. 5d), or the inhibition of autophagosome–lysosome fusion using either chloroquine or bafilomycin A_1_ (Fig. 5a). Importantly, we also observed no effect on SM-induced death with the inhibition of LC3-associated phagocytosis using *RUBCN* silencing. These data suggest that neither fully formed autophagosomes nor autophagy-mediated degradation of cargo is required to induce cell death in response to SM, as, if this were the case, a similar effect on cell death would occur regardless of autophagy stage inhibited.

**Fig. 6:**
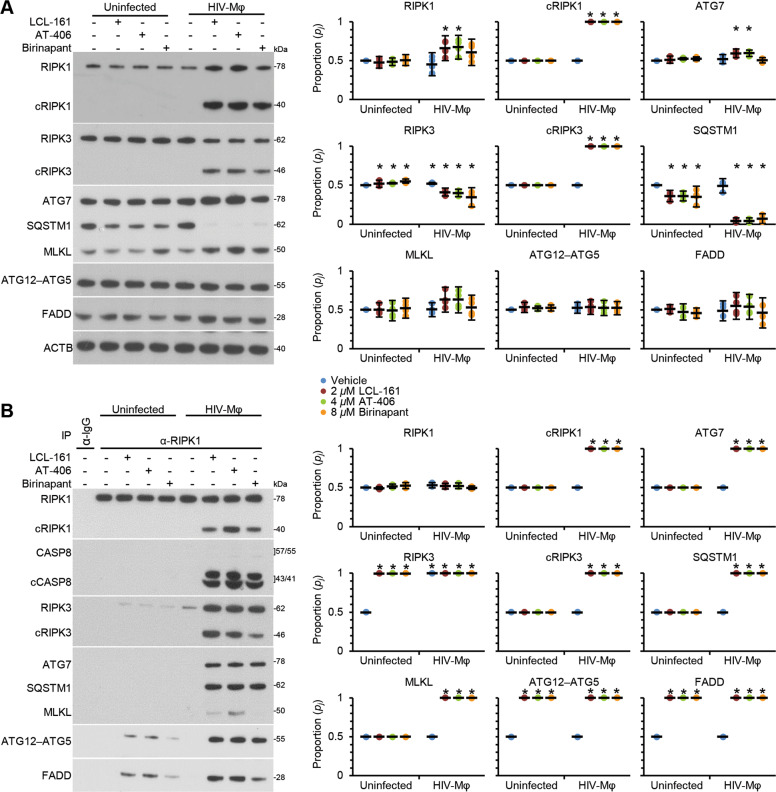
DIABLO/SMAC mimetics induce apoptosis via the formation of a caspase 8-activating platform. **a** Uninfected macrophages and HIV-Mφ were treated with 2 µM LCL-161, 4 µM AT-406, 8 µM birinapant, or vehicle for 48 h. Left, representative western blots of ATG7, ATG12–ATG5, FADD, MLKL, RIPK1, cleaved RIPK1 (cRIPK1), RIPK3, cleaved RIPK3 (cRIPK3), and SQSTM1. Right, densitometric analysis of blots *n* = 4. **b** Uninfected macrophages and HIV-Mφ were treated with 2 µM LCL-161, 4 µM AT-406, 8 µM birinapant, or vehicle for 48 h. Cells were lysed and RIPK1 was immunoprecipitated (IP). The presence of ATG7, ATG12–ATG5, FADD, MLKL, cRIPK1, RIPK3, cRIPK3, and SQSTM1 and CASP8 was assayed by western blot. Left, representative western blots. Right, densitometric analysis of blots, *n* = 4.

We next tested whether varying expression of key components of the apoptotic and necroptotic response might contribute to the differential response to SM in uninfected macrophages and HIV-Mφ. We observed no increase in the expression of RIPK1, RIPK3, FADD, MLKL, ATG5, or ATG7 in HIV-Mφ (Fig. 6a), indicating that HIV-Mφ cell death in response to SM correlates with HIV-induced increased expression of LC3B-II, BECN1, XIAP, and BIRC2. In the next series of experiments, we assessed the effect of SM on these same key players of the apoptotic and necroptotic response. At the doses tested, we observed no significant changes in the cleavage of RIPK1 or RIPK3, or the expression of ATG7, MLKL, ATG12–ATG5, or FADD in uninfected macrophages. Likewise, we did not observe an increase in ATG12–ATG5 or FADD expression in HIV-Mφ. Conversely in HIV-Mφ, we observed the SM-mediated cleavage of RIPK1 and RIPK3 (Fig. 6a), which is consistent with RIPK1 and RIPK3 being targets of CASP8 cleavage, and thus as CASP8 is activated, RIPK1 and RIPK3 are cleaved^37^. We also observed an increase in the expression of ATG7, MLKL, and FADD in HIV-Mφ, suggesting that components of the apoptotic necroptotic and autophagic machinery are involved.

### SM-induced apoptosis is dependent upon the autophagy machinery

Following the loss of BIRC2, a death-inducing signaling complex (DISC) involving RIPK1, CASP8, and FADD forms^19,38,39^. Having observed that the induction, but not the completion of autophagy is required for SM-mediated killing of HIV-Mφ, we examined whether the autophagy machinery might provide a scaffold for the formation of a DISC using co-immunoprecipitation. While the DISC proteins, RIPK3 and FADD co-immunoprecipitated with RIPK1 in uninfected macrophages and HIV-Mφ, CASP8 and MLKL only co-immunoprecipitated with RIPK1 in HIV-Mφ (Fig. 6b). Also in HIV-Mφ, proteins involved in early-to-mid stages of autophagy including ATG5, ATG7, and SQSTM1 co-immunoprecipitated with RIPK1 (Fig. 6b), while proteins that are important for earlier signaling in the autophagy process (BECN1 and ULK1 (unc-51-like autophagy-activating kinase 1)) and those involved in later steps, when autophagosomes fuse with lysosomes (syntaxin 17 [STX17] and lysosomal-associated membrane protein 1 (LAMP1)) did not.

**Fig. 7:**
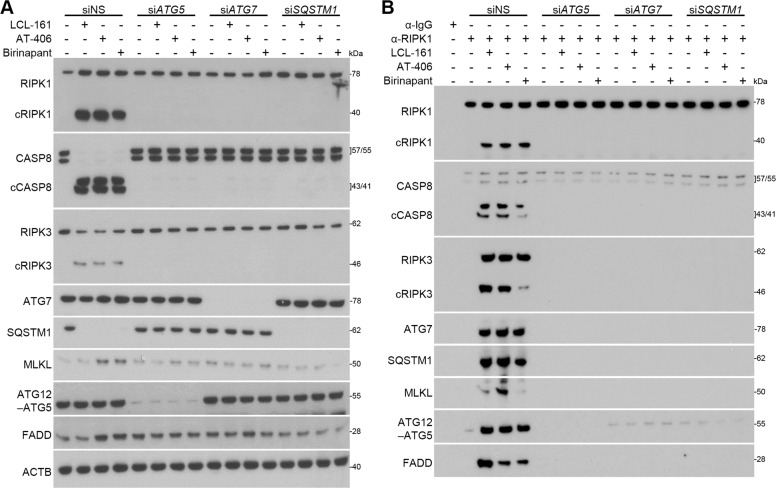
DIABLO/SMAC mimetics induce apoptosis via an autophagy-dependent caspase 8 activating platform. HIV-Mφ transfected with *ATG*_*5*_ siRNA (*siATG*_*5*_), *ATG*_*7*_ (*siATG*_*7*_), *SQSTM*_*1*_ (*siSQSTM*_*1*_), or scrambled shRNA (shNS) were treated with 2 µM LCL-161, 4 µM AT-406, 8 µM birinapant, or vehicle for 48 h. **a** Representative western blots of ATG7, ATG12–ATG5, FADD, MLKL, RIPK1, cleaved RIPK1 (cRIPK1), RIPK3, cleaved RIPK3 (cRIPK3), and SQSTM1, *n*=4. Densitometric analysis of blots is shown in Supplementary Fig. 2. **b** Cells were lysed and RIPK1 was immunoprecipitated (IP). The presence of ATG7, ATG12–ATG5, FADD, MLKL, cRIPK1, RIPK3, cRIPK3, and SQSTM1 and CASP8 in immunoprecipitates was assayed by western blot, *n* = 4. Densitometric analysis of blots is shown in Supplementary Fig. 3.

We next assessed whether the autophagy proteins ATG5 and ATG7 are required for efficient SM-mediated DISC formation and activation in HIV-Mφ using RNAi. As RIPK1 directly interacts with SQSTM1 (ref. ^40^), we also silenced for *SQSTM1*. Silencing for *ATG5*, *ATG7*, or *SQSTM1* each ablated the SM-mediated CASP8, RIPK1, and RIPK3 cleavage (Fig. 7a, Supplementary Fig. 2) and induced a significant negative change in CASP8, RIPK3, MLKL, ATG7, ATG5, FADD, and SQSTM1 co-immunoprecipitation with RIPK1 (Fig. 7b, Supplementary Fig. 3). Notably, MLKL did not associate with RIPK1 after any RNAi. These findings correspond to the increase in cell viability observed upon SM treatment of *ATG5*, *ATG7*, and *SQSTM1*-silenced cells (Fig. 5d). Collectively, these data indicate that SM require ATG5, ATG7, and SQSTM1 for the assembly and activation of a DISC in HIV-Mφ.

**Fig. 8:**
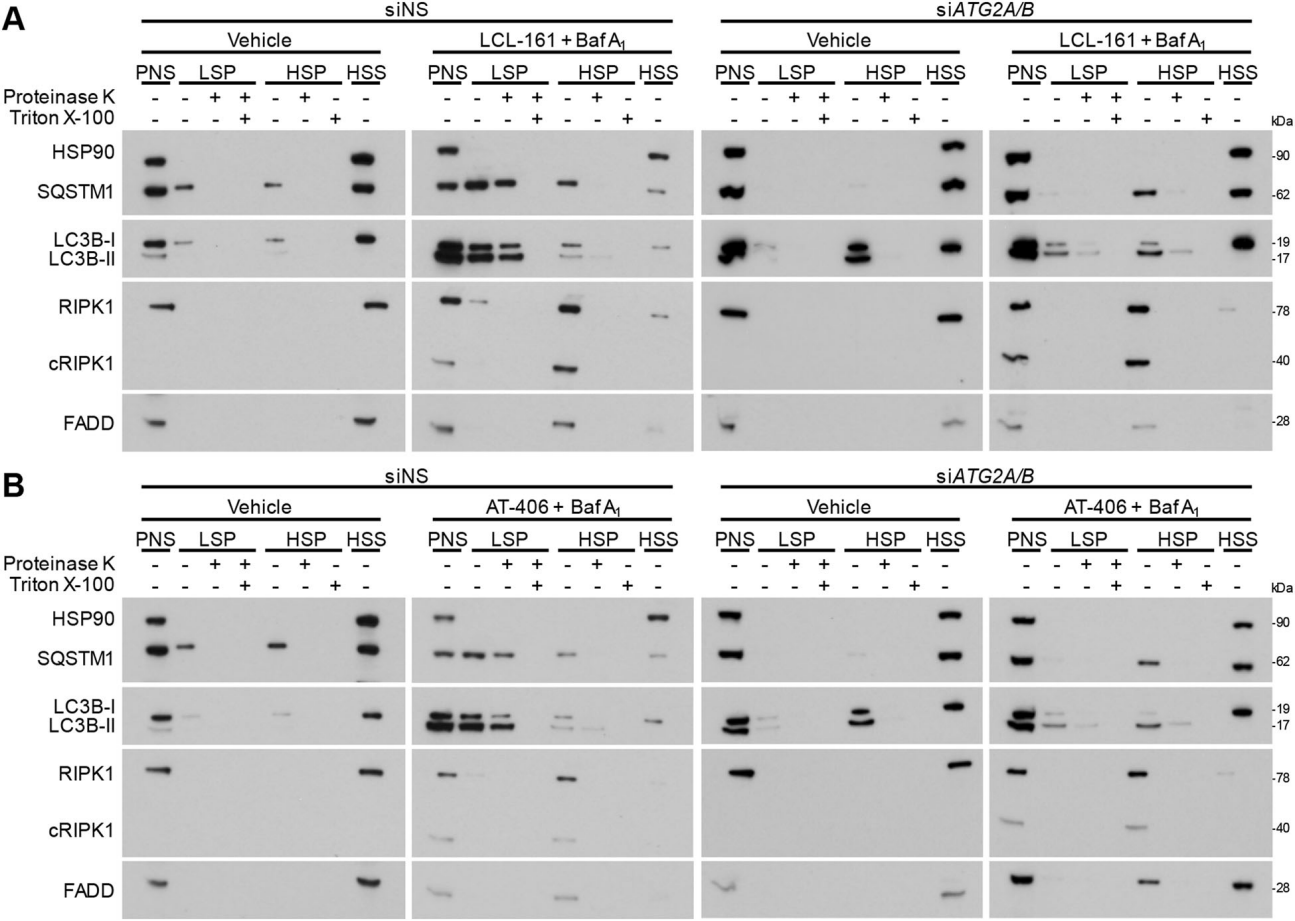
DIABLO/SMAC mimetics induce the formation of a caspase 8 activating platform using phagophore membranes as a scaffold. HIV-Mφ simultaneously transfected with a mixture of siRNA against *ATG2A* and *ATG2B* (si*ATG2A/B*) or scrambled siRNA (siNS) were cultured in growth media supplemented with both 2 µM LCL-161 (**a**) or 4 µM AT-406 and 200 nM bafilomycin A_1_ (Baf A_1_) or vehicle for 2 h. The post nuclear supernatant (PNS) was separated into a LSP, a HSP, and a HSS fraction, and subjected to western blotting. The subfractions were treated with proteinase K with or without Triton X-100. Representative western blots are shown, *n* = 4. Analysis of silencing is shown in Fig. 5c.

As RIPK1 directly interacts with SQSTM1, it is possible that DISC components translocate to autophagosomal membranes. Therefore, to determine the stage of autophagosome formation at which DISCs localize to autophagic structures, we analyzed membranes in *ATG2A*/*B*-silenced cells using differential centrifugation, using LC3B-II and SQSTM1 as markers of autophagosome membranes and autophagy substrates, respectively^41^. In the control cells, and in the absence of SM, LC3B (as LC3B-I), SQSTM1, FADD, and RIPK1 were located primarily in the cytosol (the high-speed supernatant (HSS) fraction; Fig. 8). However, in the presence of SM and bafilomycin A_1_, LC3B and SQSTM1accumulated in the low-speed pellet (LSP) and was resistant to proteinase K but sensitive to Triton X-100, indicating that they were enclosed in autophagosomes. Conversely, SQSTM1, RIPK1, cleaved RIPK1, and FADD accumulated in the high-speed pellet (HSP) that was sensitive to both proteinase K and Triton X-100, indicating that these were not enclosed structures. In *ATG2A*/*B*-silenced cells proteinase K-sensitive LC3B-II and SQSTM1 accumulated in the HSP fraction in the absence of birinapant and bafilomycin A_1_. In the presence of SM and bafilomycin A_1_, LC3B-II and SQSTM1 accumulated in both the LSP and HSP fractions, while proteinase K-sensitive RIPK1, cleaved RIPK1, and FADD were present only in the HSP fraction (Fig. 8). Collectively, this suggests that ATG2A and ATG2B are required for complete closure of autophagosomes, as LC3B-II-positive structures accumulate in their absence^41^, and that RIPK1 and FADD do not translocate into closed autophagosomes after SM treatment.

## Discussion

Although ART has greatly improved life expectancy and quality for those infected with HIV, multidrug resistance continues to increase, emphasizing the importance of identifying novel strategies to improve HIV treatment with the goal of reducing the size of the viral reservoir. Although current efforts to purge the latent HIV reservoir predominantly endeavor to reactivate viral production from latently infected CD4+ T cells followed by immunologic suppression or viral clearance through ART, this strategy has, to date, failed to decrease significantly the viral reservoir^42–46^. This approach will be even less effective in macrophages since HIV-infected macrophages are particularly resistant to HIV-mediated apoptosis and CD8+ T-cell-mediated killing^8–10^. Despite the documented importance of macrophages, few studies have targeted killing HIV-infected macrophages as part of a cure strategy, although some show promise^21,47–49^.

In the present study, we used an alternative strategy to activate cell death pathways selectively in HIV-infected macrophages, while sparing uninfected cells. Similar to our data with HIV-T_CM_^19^, the selective SM-mediated apoptosis of HIV-Mφ is dependent upon the induction of autophagy and the translocation, and interaction of FADD and the CASP8 homocomplex with both ATG5 and SQSTM1—self-oligomerized SQSTM1 localizes and recruits RIPK1 to the endoplasmic reticulum-associated autophagosome formation site independently of LC3 and is retained as autophagosomes mature^50,51^, while CASP8 and RIPK1 tethering to autophagosomal membranes involves ATG5 and FADD^52^. We also show that in HIV-Mφ, RNAi and pharmacological inhibition of early autophagy events inhibits the SM-mediated formation of a cytosolic DISC, CASP8 activation, and subsequent cell death, whereas neither simultaneous silencing of *ATG2A* and *ATG2B*, nor the pharmacological inhibition of autophagosome–lysosome fusion protected cells from SM-mediated cell death. Moreover, RIPK1 and FADD co-localize with SQSTM1 in the HSP. Collectively these data suggest that autophagy proteins serve as a platform for the assembly of a cytosolic DISC that initiates apoptosis, linking autophagy to the control of cell survival and death.

Importantly, two of the SM tested, LCL-161 and birinapant, failed to increase the viral production from HIV-Mφ. These findings correlate with previous studies that showed that the SM SBI-0637142, LCL-161, GDC-0152, and birinapant all failed to activate latent provirus in resting CD4+ T cells collected from HIV-infected patients undergoing ART^19,53^. Conversely, AT-406 increased the production of HIV p24 from HIV-Mφ in agreement with Bobardt et al.^54^. This increase correlated with increased TNF production not observed with either LCL-161 or birinapant treatment.

During HIV infection, macrophages are primary producers of TNF, express constitutively activated NFκB, upregulate IAPs, and are resistant to many apoptotic stimuli, including TNF superfamily members^55,56^. In this state, binding of TNF to its receptor leads to the activation of TNF complex I, comprised of TRADD, the ubiquitin ligases TRAF2 and TRAF5, IKBKG (NEMO), and TNF alpha-induced protein 3 together with the linear ubiquitin chain assembly complex, RIPK1, BIRC2, and BIRC3. In addition to binding caspases, BIRC2 also interacts with TRAF2, which mediates their recruitment to TNFRSF1A. The BIR3 domain of BIRC2 binds RIPK1, while its E3 ligase activity mediates the ubiquitination of RIPK1, stimulating NFκB activation and suppressing activation of CASP8, ultimately leading to pro-survival signaling^25,57,58^. After SM-induced degradation of IAPs, the TNF-receptor ligation initiates the formation of a DISC known as TNF complex IIa, which leads to the cleavage and activation of CASP8 and the induction of apoptosis, resulting in the reversal of the role of TNF from promoting survival to inducing cell death^59^. The evidence for this occurring in SM-treated HIV-Mφ stems from the TNF neutralization experiment that demonstrated that neutralizing TNF completely ablated the effect of SM, indicating the dependency of SM-mediated apoptosis of HIV-Mφ on TNF (Fig. 2e). This is in contrast to what we found in HIV-T_CM_ where SM-mediated apoptosis was independent of TNF^19^. However, despite the initiation of apoptosis and the involvement of caspases, we were unable to block cell death using the pan-caspase inhibitor z-VAD-FMK. In this case, when we reduced IAP activity through treatment with SM while inhibiting caspases, MLKL became activated, and the cells died through caspase-independent necroptosis (Fig. 5b)^30^. Therefore, SM can potentiate TNF-stimulated cell death of HIV-Mφ by promoting either apoptosis or necroptosis in a context-dependent manner. Thus, for the purposes of sensitizing HIV-Mφ to the cytotoxic effects of HIV, IAPs are critical targets.

In summary, our data identify a molecular mechanism whereby we can selectively induce apoptosis in HIV-infected macrophages through a SM-mediated autophagy-dependent assembly of a DISC. Our study thus highlights IAPs as therapeutic targets to clear the macrophage HIV reservoir, and suggests that current HIV cure strategies should include the targeting of IAPs in efforts to achieve HIV eradication.

## Materials and methods

### Macrophages

Venous blood was drawn from human subjects using protocols approved by the Human Research Protections Program of the University of California San Diego in accordance with the requirements of the Code of Federal Regulations on the Protection of Human Subjects (45 CFR 46 and 21 CFR 50 and 56). All volunteers gave written informed consent prior to their participation. PBMC were isolated from whole blood by density gradient centrifugation over Ficoll-Paque Plus (GE Healthcare). Monocyte derived macrophages were prepared from PBMC by adherence, using colony stimulating factor 1 (Peprotech) as previously described^21^.

### HIV

The following were obtained from the NIH AIDS Reagent Program: HIV_Ba-L_ from Suzanne Gartner, Mikulas Popovic, and Robert Gallo^60,61^ and HIV_93IN905_ from Kavita Lole, Robert Bollinger, and Stuart Ray^62^. Virus stocks were prepared as previously described^19,63^. Macrophages were infected with HIV at 0.04 multiplicity of infection for 4 h, washed then cultured for 10 days before use in experiments as previously described^21,64^.

### Cell death assays

Apoptotic ssDNA damage measurements were made using the ssDNA Apoptosis ELISA Kit (Millipore) as described previously^65^. LDH activity in cell supernatants was measured using a mixture of diaphorase/NAD^+^ and 3-(4-iodophenyl)-2-(4-nitrophenyl)-5-phenyl-2H-tetrazol-3-ium chloride/sodium 2-hydroxypropanoate using the LDH^PLUS^ assay (Roche). Percent cytotoxicity was calculated according to the manufacturer’s protocol using a LDH standard curve and a cell lysate standard curve.

### Chemicals

LCL-161, AT-406, birinapant (Selleck Chemicals) and staurosporine (Sigma) were prepared in dimethyl sulfoxide. Corresponding volumes of DMSO (5 µL DMSO mL^−1^) were used for vehicle in each experiment. For inhibitor experiments, 20 µM z-VAD-FMK, 200 nM wortmannin, 10 µM chloroquine, 10 nM bafilomycin A_1_, or 10 µM necrostatin-1 (all Sigma) were added for 1 h prior to, and for the duration of the experiment. The human TNF neutralizing antibody (D1B4; Cell Signaling) was added 2 h prior to SM treatment at 1 µg mL^−1^ as previously described^19^.

### siRNA transfection

Macrophages were transfected with ThermoFisher Silencer Select *ATG2A* (ID# s23098), *ATG2B* (ID# s30173), *ATG5* (ID# s18159), *ATG7* (ID# s20651), *RUBCN* (ID# s18718), *SQSTM1* (ID# s16961), *ULK1* (ID# s15965), or control (Cat# 4390846) siRNA (siNS), using lipofectamine RNAiMAX transfection reagent (ThermoFisher) in Opti-MEM (Gibco) according to the manufacturer’s instructions. Forty eight hours later, cells were analyzed for target gene silencing and used in experiments. Transfection efficiency was assessed with BLOCK-iT Alexa Fluor Red Fluorescent Control (ThermoFisher) using flow cytometry^19^.

### Western blotting

ATG2A (#15011), ATG5 (#2630), ATG7 (#2631), BECN1 (#3738), BIRC2 (D5G9), CASP8 (1C12), FADD (#2782), FAS (C18C12), FASLG (#4273), HSP90 (C45G5); LAMP1 (C54H11), MLKL (D2I6N), PARP1 (46D11), RIPK1 (D94C12), RIPK3 (D4G2A), ULK1 (D9D7), and XIAP (3B6) antibodies were obtained from Cell Signaling. SQSTM1 (#ab56416) and STX17 (#ab116113) antibodies were purchased from Abcam. *β*-actin (ACTB; AC-74) and ATG2B (#HPA019665) were purchased from Sigma. MAP1LC3B (#NB100-2220) was purchased from Novus Biologicals. Cell lysis, co-immunoprecipitation, subcellular fractionation, and western blotting were performed as previously described^19,41^. Relative densities of the target bands to the reference bands was calculated using ImageJ (NIH).

### Statistics

Samples were assigned to experimental groups through simple random sampling. Sample size (*n*) was determined using a two-sample two-sided equality test with power (1 − *β*) = 0.8, *α* = 0.05, and preliminary data where the minimum difference in outcome was at least 70%. Data are represented as dot blots with arithmetic means ± 95% confidence interval for independent biological replicates. Data were assessed for symmetry, or skewness, using Pearson’s skewness coefficient. Normalized ratiometric data were log_2_ transformed. When expression in the reference sample is low or zero, we used proportion statistics as described previously^19,66^. Comparisons between groups were performed using the paired, two-tailed, Student’s *t*-test. In all experiments, differences were considered significant when *P* < 0.05; *P < 0.05.

## Supplementary information

Supplementary Information

Supplementary Figure S1

Supplementary Figure S2

Supplementary Figure S3
