# Supplementary material for: SMAC mimetics induce autophagy-dependent apoptosis of HIV-1-infected macrophages
Source: Cell Death Dis. 2020 Jul 27;11(7):590. doi: 10.1038/s41419-020-02761-x (PMC7385130; doi:10.1038/s41419-020-02761-x)
Supplement: Supplementary file 1 — Supplementary Information [file 41419_2020_2761_MOESM1_ESM.docx]

**Supplemental Information**

**SMAC mimetics induce autophagy-dependent apoptosis of HIV-1-infected macrophages**

Grant R. Campbell, Rachel K. To, Gang Zhang, and Stephen A. Spector

**SUPPLEMENTAL FIGURE LEGENDS**

**Figure S1.**

Related to Figure 1. Macrophages were left uninfected or infected with HIV for 10 days after which cells were harvested, stained for HIV p17 expression, and analyzed by flow cytometry. Representative histogram plots shown, *n* =3.

**Figure S2.**

Related to Figure 7A. HIV-Mφ transfected with *ATG5* siRNA (si*ATG5*), *ATG7* (si*ATG7*), *SQSTM1* (si*SQSTM1*) or scrambled shRNA (shNS) were treated with 2 *µ*M LCL-161, 4 *µ*M AT-406, 8 *µ*M birinapant, or vehicle for 48 h. Cells were lysed and resolved by western blot. Representative western blots of ATG7, ATG12–ATG5, FADD, MLKL, RIPK1, cleaved RIPK1 (cRIPK1), RIPK3, cleaved RIPK3 (cRIPK3), and SQSTM1 are shown in Figure 7A. Densitometric analysis of blots is shown here. *n* = 4.

**Figure S3**

Related to Figure 7B. HIV-Mφ transfected with *ATG5* siRNA (si*ATG5*), *ATG7* (si*ATG7*), *SQSTM1* (si*SQSTM1*) or scrambled shRNA (shNS) were treated with 2 *µ*M LCL-161, 4 *µ*M AT-406, 8 *µ*M birinapant, or vehicle for 48 h. Cells were lysed and and RIPK1 was immunoprecipitated (IP). Representative western blots of ATG7, ATG12–ATG5, FADD, MLKL, RIPK1, cleaved RIPK1 (cRIPK1), RIPK3, cleaved RIPK3 (cRIPK3), and SQSTM1 are shown in Figure 7B. Densitometric analysis of blots is shown here. *n* = 4.
