# Supplementary figures and images for: SMAC mimetics induce autophagy-dependent apoptosis of HIV-1-infected macrophages
Source: Cell Death Dis. 2020 Jul 27;11(7):590. doi: 10.1038/s41419-020-02761-x (PMC7385130; doi:10.1038/s41419-020-02761-x)

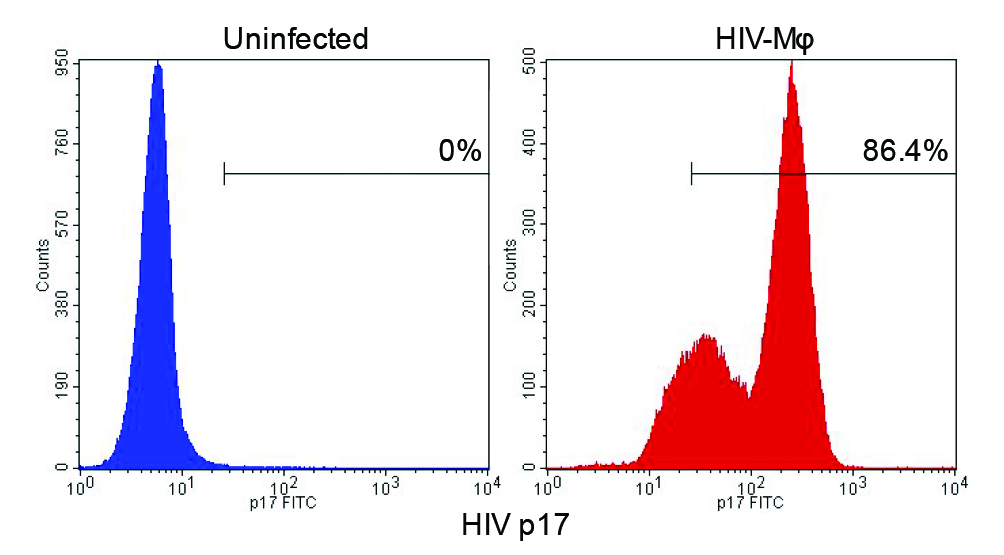

Supplement: Supplementary file 2 — Supplementary Figure S1 [file 41419_2020_2761_MOESM2_ESM.tif]

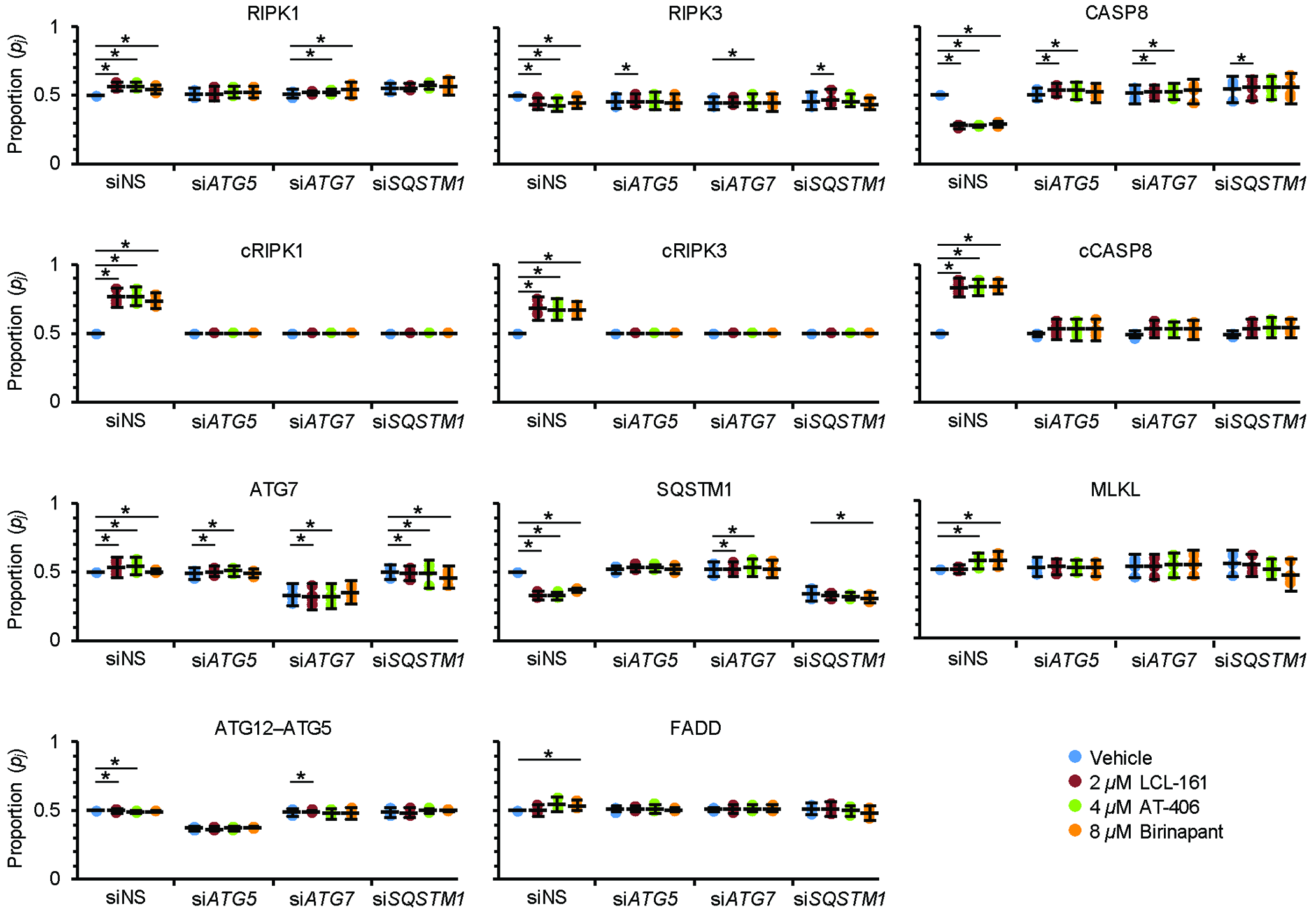

Supplement: Supplementary file 3 — Supplementary Figure S2 [file 41419_2020_2761_MOESM3_ESM.tif]

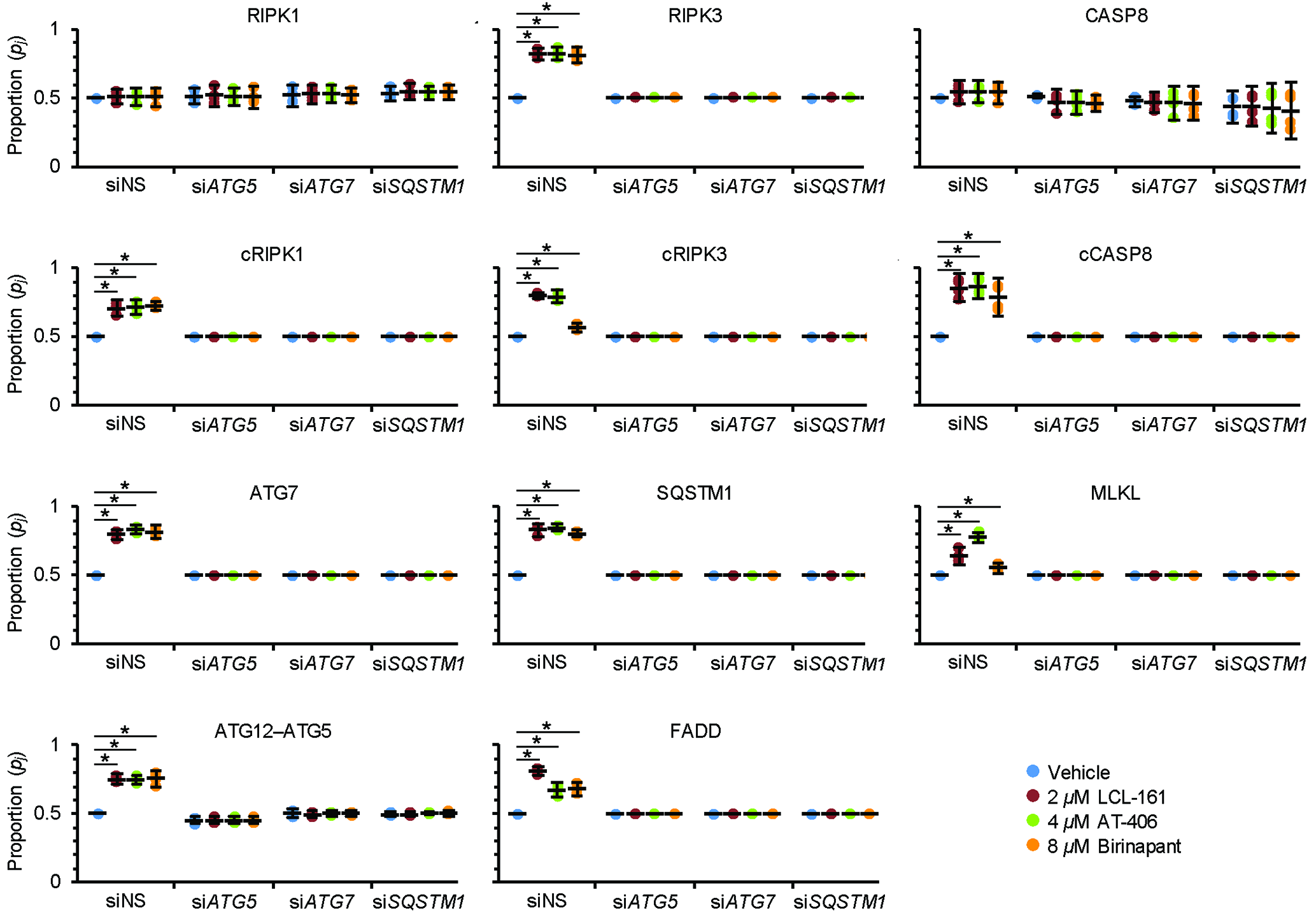

Supplement: Supplementary file 4 — Supplementary Figure S3 [file 41419_2020_2761_MOESM4_ESM.tif]
